# Alveolar cleft reconstruction using autogenous double iliac corticocancellous bone blocks technique versus particulate autogenous spongy bone graft from anterior iliac crest

**DOI:** 10.1007/s00784-025-06288-3

**Published:** 2025-04-15

**Authors:** Louai Raafat, Mohammed Omara, Ragia Mounir

**Affiliations:** Oral and Maxillofacial Surgery Department, Faculty of Dentistry, Cairo University, Cairo, Egypt

**Keywords:** Secondary alveolar cleft bone grafting, Phase of mixed dentition, Anterior iliac crest, Iliac cancellous bone graft, Double iliac corticocancellous bone blocks

## Abstract

**Objective:**

The primary goals for alveolar cleft grafting are to gain and maintain bone in the cleft area that provides continuity for the maxillary segments, allowing the stability of the maxilla and building the bony foundation for the erupting cleft teeth and closure of the oronasal communication. The current study compared the effectiveness of the double iliac corticocancellous bone blocks technique versus the particulate autogenous spongy bone graft from the anterior iliac crest in alveolar cleft grafting in the mixed dentition stage.

**Patients and methods:**

The current randomized clinical study included 18 patients with unilateral alveolar clefts. They were divided into two equal groups according to the technique used for grafting; group (1) included nine patients in whom grafting with double iliac corticocancellous bone blocks with cancellous bone particulates in between was used (study group), and group (2) included nine patients in whom conventional cancellous particulate bone grafting from anterior iliac crest was used (Control group).

**Results:**

Nine months postoperatively, the study group showed superior results regarding graft width, height, and volume compared to the control group in the current study.

**Conclusion:**

Regarding the graft success factors represented by the maintained graft labio-palatal width, graft height, and total graft volume, the technique of double iliac corticocancellous bone blocks was markedly effective in reconstructing alveolar clefts when compared with the conventional grafting technique that utilized cancellous particulate bone alone from the anterior iliac crest.

Clinical relevance.

The double iliac corticocancellous bone blocks technique maintained the grafted bone volume, width, and height.

## Introduction

Alveolar cleft is a congenital anomaly of the dentoalveolar complex presenting itself as a tornado-shaped bone defect in the maxilla, usually in the area of the lateral incisor and canine [1, 2]. Various sources of bone can be utilized for alveolar cleft grafting: either autogenous bone from different donor sites such as anterior and posterior iliac crest, mandibular bone, rib, cranial bone, and tibia, or other options of graft material, including the availability of synthetic, xenogenic, and allogenic bone. However, utilizing autogenous bone from the anterior iliac crest remains the most dependable method and is considered the gold standard due to its ease of accessibility and abundant amounts of autogenous bone that contain viable cells and growth factors promoting osteogenic activity [1–8].

Alveolar cleft grafting main objectives are, restoring the continuity of the alveolus and maxilla and hence maintaining the stability of the maxillary arch, building the bony foundation for either the erupting teeth in the cleft area, or for the future implant replacement of the missing teeth, preserving the health of the periodontium of the already erupted teeth adjacent to the cleft, supporting the lip and the base of the ala of the nose, closure of the oronasal fistula, and accommodating for any tooth movement around the cleft during orthodontic treatment [1, 2, 8].

To achieve these objectives, the graft should maintain a bone bridge with acceptable labio-palatal thickness, height, and acceptable volume. Since introducing the concept of secondary alveolar bone grafting (SABG) by **Boyne** and **Sands**, which utilized spongy/cancellous bone from the anterior iliac crest, many studies have been published confirming the technique's success [9]. Hence, the technique was considered the gold standard. However, most of these studies utilized scores and scales like the Enemark score, and Bergland and Chelsea scales in their evaluation. These scales depended on two-dimensional radiographs such as occlusal and panoramic X-rays to assess only the graft height level [7]. It was thought that this radiographic evaluation usually overestimated the graft success and neglected the other aspects of graft success criteria, like labio-palatal thickness, which is more important in deciding whether the cleft grafting objectives have been fulfilled [5, 8–21].

Recent studies found that it was more convenient to rely on 3D imaging methods, such as CT and CBCT, in following the changes in graft height, volume, and width after surgery to assess if the grafting objectives were achieved or not, in the assessment of the cleft and the surrounding dentition and to estimate the average amount of graft needed before surgery [8–21]. Recent studies that followed the changes in the graft labio-palatal width and/or graft volume after SABG using a particulate cancellous bone graft from the anterior iliac crest found that in many cases, neither the width nor the volume of the graft was maintained, and the graft suffered from reduction in volume, width and even in height to some degree, these findings pushed the cleft care providers to develop various grafting maneuvers to maintain the grafted bone width, height, and volume during graft healing [8–21].

Many trials were conducted to improve graft stability and to reduce its resorption. Those trials included covering the graft with a resorbable membrane, utilizing Ti-meshes for graft support, using the mineralized plasmatic matrix (MPM), or using cortical piece/s of bone either from symphysis, external oblique ridge in older patients or from the ilium to support the cancellous graft either by using it as a ceiling to the graft (under the nasal mucosa) or by adding this cortical piece of bone at the palatal side alone or at the buccal side alone or the use of double corticocancellous bone blocks overlapping the palatal and labial sides of the cleft with cancellous bone in between [8, 22–34].

The current study aimed to compare the double iliac corticocancellous bone blocks technique with cancellous bone in between versus the conventional grafting technique of the cleft using spongy bone derived from the anterior iliac crest to find out which one maintained graft width, height, and volume after the healing period and hence fulfilled the objectives of grafting the alveolar cleft.

### Patients and methods

#### Study design

The current study was a single-blinded, parallel, randomized controlled clinical trial. The study was conducted at the Faculty of Dentistry, Oral and Maxillofacial Surgery Department, Cairo University. The study was registered on ClinicalTrials.gov at March 7, 2022 under the registry number NCT05283005. It adhered to the Declaration of Helsinki on medical research ethics and received approval letter from the Institutional Research Ethics Committee of the Faculty of Dentistry, Cairo University (IRB number: 29 12 21).

#### Intervention and participants

This study aimed to compare two different grafting techniques for reconstruction of alveolar cleft defects in patients with unilateral alveolar cleft, the double iliac corticocancellous bone blocks with interspaced cancellous bone grafting technique as a study group versus the conventional autogenous iliac cancellous bone grafting technique as a gold standard control group. Patients with unilateral alveolar cleft were selected based on the following clinical criteria, patients with unilateral maxillary alveolar cleft at the mixed dentition phase (between the ages of 7 and 13 years old) from both sexes; males and females, patients whom cleft lip and palate were repaired, patients who didn’t receive any previous surgery for grafting of the alveolar cleft, and patients with proper oral hygiene were included in the study. However, patients with poor oral hygiene, active infection and/or periodontitis, uncontrolled diabetes, or immunocompromised weren’t included in the study.

#### Outcomes

Primary outcome was to evaluate the bone height and width of the graft immediately after surgery versus 9 months postoperatively in the two groups, while the secondary outcome was to evaluate the graft volume immediately after surgery versus 9 months postoperatively in the two groups and compare these changes between the two groups to evaluate graft stability during the healing period.

#### Sample size calculation

A power analysis was designed to have adequate power to apply a two-sided statistical test of the null hypothesis that there is no difference between tested groups. By adopting an alpha level of (0.05) a beta of (0.2) i.e. power = 80% and an effect size (d) of (1.85) calculated based on the results of *Omara, Mohammed, *et al. [33] the predicted sample size (n) was found to be (12) cases. The sample size was increased by (25%) to account for any possible dropouts to be (16) cases. Extra (2) cases were then also added, one in each group to be (18) cases. Sample size calculation was performed using PS Power and Sample Size Calculations version 3.1.2 (Dupont WD, Plummer WD: "Power and Sample Size Calculations: A Review and Computer Program", Controlled Clinical Trials 1990; 11:116–28.)

#### Preoperative preparation

All participants were recruited from the out-patient clinic of the Faculty of Dentistry Cairo University. Recruited patients were inspected, examined, and evaluated for previous and current medical and dental history to identify eligible patients to undergo surgery under general anesthesia. Preoperative CBCT for the maxilla was requested from the selected 18 patients as a baseline record, for preoperative cleft volume measurements for both groups using 3D Slicer software, and as a primary survey to exclude if any lesion was present within the cleft area that might make the patient ineligible for the study and to detect any impacted tooth in the cleft area. The CBCT was limited to the maxilla and midface and was obtained using the machine (Planmeca ProMax 3D Classic, Planmeca, Finland). The exposure parameters were 85 kVp, 15 Ma, and 6 cm FOV. Informed consents were signed by patients’ parents or guardians after an explanation of the treatment plan, side effects, and any possible complications. Pre-surgical initial orthodontics were carried out to patients who were found with severely misaligned central incisors to allow for better access for graft placement and soft tissue closure.

#### Patients randomization

The selected and prepared 18 patients were randomly allocated into two equal groups using a simple random sequence with an allocation ratio of 1:1 produced by the website (www.random.org). Group (A): 9 cleft patients were treated with the double iliac corticocancellous bone blocks with cancellous bone particulates in between (Study group). Group (B): 9 cleft patients were treated with the conventional technique (Control group).

#### Surgical procedure

All patients were treated under general anesthesia with nasotracheal intubation. In both groups, injection of Articaine (ARTINIBSA 4% 1:100,000®) into both the recipient site (alveolar cleft site) and the donor site (anterior iliac crest site) was made to control postoperative pain and for hemostasis. For both groups, the labial mucosa was incised along the cleft margin with elliptical incisions around the labial oronasal fistula. Then, on both sides of the cleft, a sulcular incision around two or three teeth on each side of the cleft was performed according to the defect size and the grafting technique, followed by two vertical diverging release incisions on both sides starting from the gingival margin to the labial vestibule followed by small anteriorly converging incisions at the end of each release incision giving the hockey stick appearance, which, together with the periosteal release of the flap, facilitated the advancement of labial mucosa and approximation toward the palatal mucosa without tension and hence allowed for easier graft coverage with soft tissue. The mucosa covering the cleft's margins was dissected and retracted, exposing the cleft’s bony margins. The palatal flap was raised using a sulcular incision on the palatal side of teeth on both sides of the cleft. Finally, nasal mucosa was also dissected, elevated, and sutured tightly for a watertight seal separating the nasal cavity and the alveolar cleft defect with the nasal layer becoming the roof of the cleft.

The anterior iliac crest and anterior iliac spine were identified so that the incision was carried out posterior to the superior iliac spine by about 1.5 cm to avoid injury to the subcostal nerve that passes over it. Also, the skin over the anterior iliac crest was retracted medially by gentle abdominal pressure so that the incision of the skin was made 2 cm lateral to the anterior iliac crest to avoid its compression or abrasion by a belt or tight waistband. The following layers were incised and dissected till the attachment between the external abdominal oblique and the tensor fascia lata muscle was identified. Then, this attachment was incised and retracted medially to expose the cartilaginous cap covering the iliac crest.

#### Control group

**Fig. 1:**
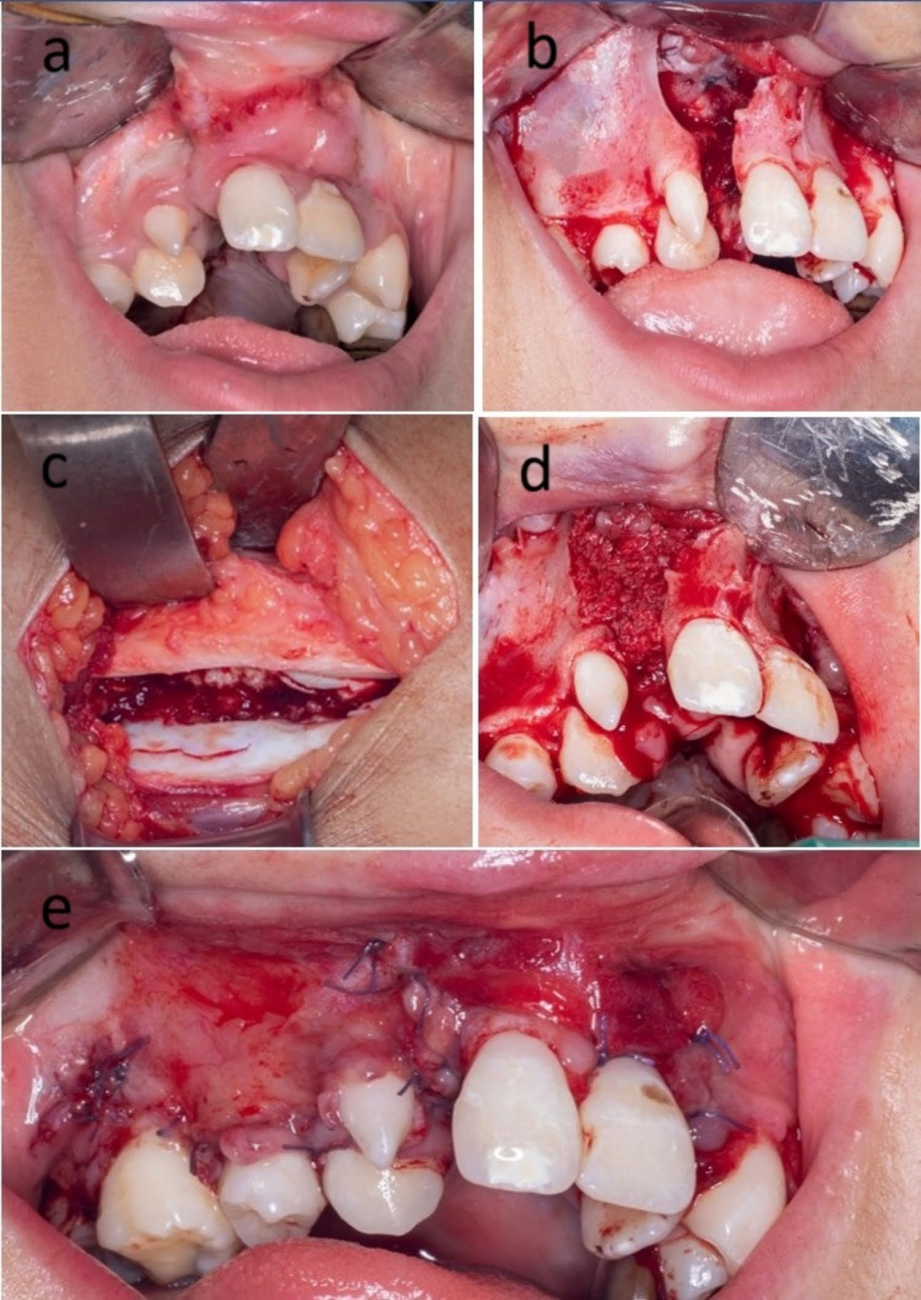
Clinical photographs showing alveolar cleft grafting using conventional cancellous bone particles derived from the anterior iliac crest. (Control group)

In the control group, the cap was incised in its middle and retracted slightly medially and laterally to expose the bone of the AIC. Once the bone of the anterior iliac crest had been accessed, cancellous bone chips were harvested using a large bone curette. Afterwards, closure of the donor site was done layer by layer. The harvested bone particles were packed within the cleft, followed by the closure of the flap (Fig. 1).

#### Study group

In the study group, after reaching the cap, a medial approach was used by reflecting the external abdominal oblique and transversus abdominis muscles and retracting them medially from the mid-crest and medial edge of the crest. The periosteal elevator was then directed downward and medially to elevate and retract the iliacus muscle to access the medial cortical side of the anterior ilium. A medial mono-cortical bony window was outlined using a micro disc for making two horizontal parallel cuts, while a round rose head or thin surgical fissure bur was used to create two vertical cuts connecting the horizontal ones and to confirm the horizontal cuts to facilitate the insertion of the spatula chisels through the cuts by which the separation of the cortical window from the underlying cancellous bone was done. After that, a large bone curette was used to harvest the cancellous bone from underneath the removed cortical window, then a hemostatic sponge was applied, and the wound closure was done layer by layer, respectively by Vicryl 4.0 (Polyglactin 910; Ethicon) for deep tissue layers and followed by closure of the skin layer by proline 4.0.

**Fig. 2:**
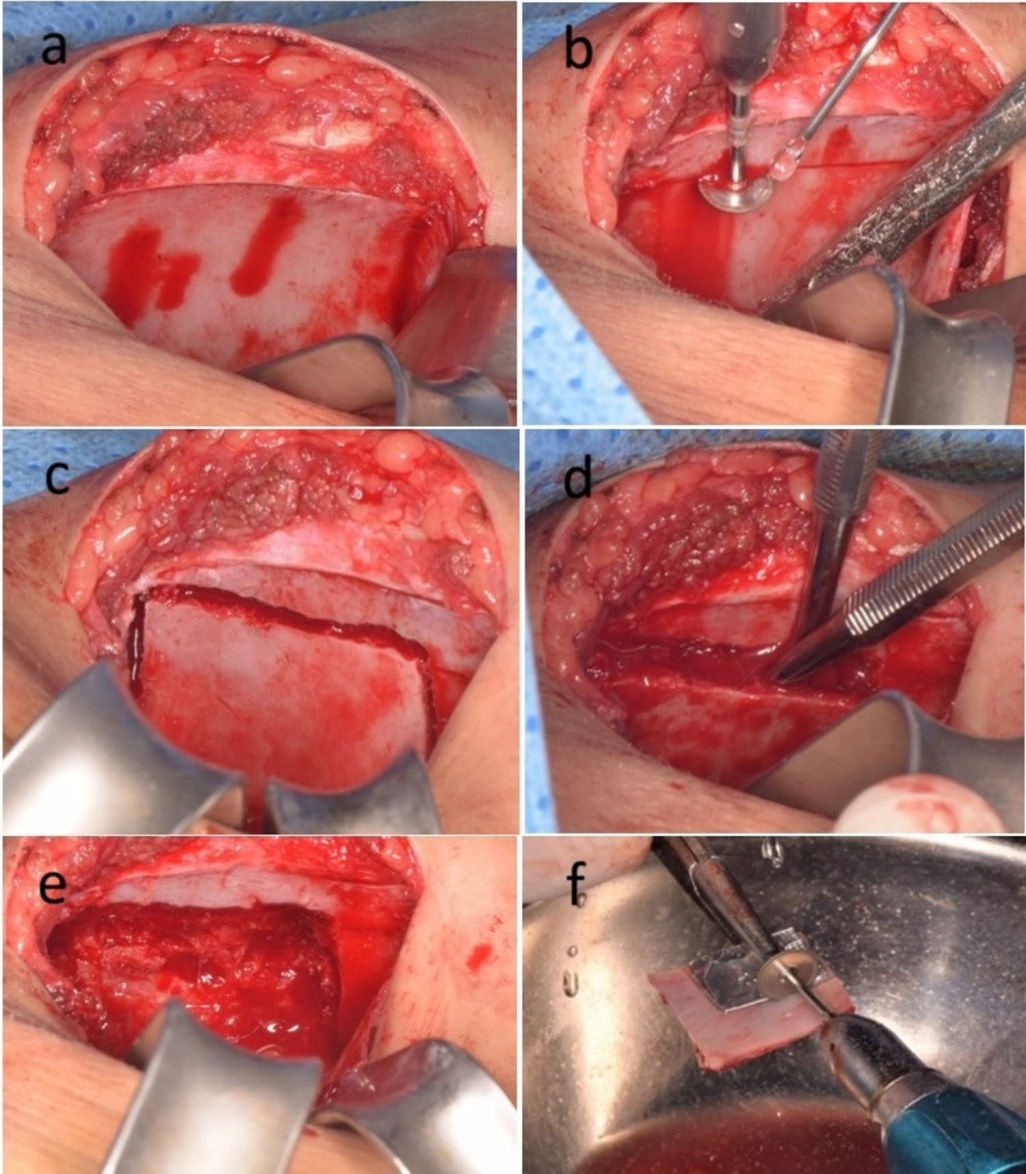
Clinical photographs showing the Medial approach to the anterior iliac crest and cortical bone block harvesting and preparation (Study group)

**Fig. 3:**
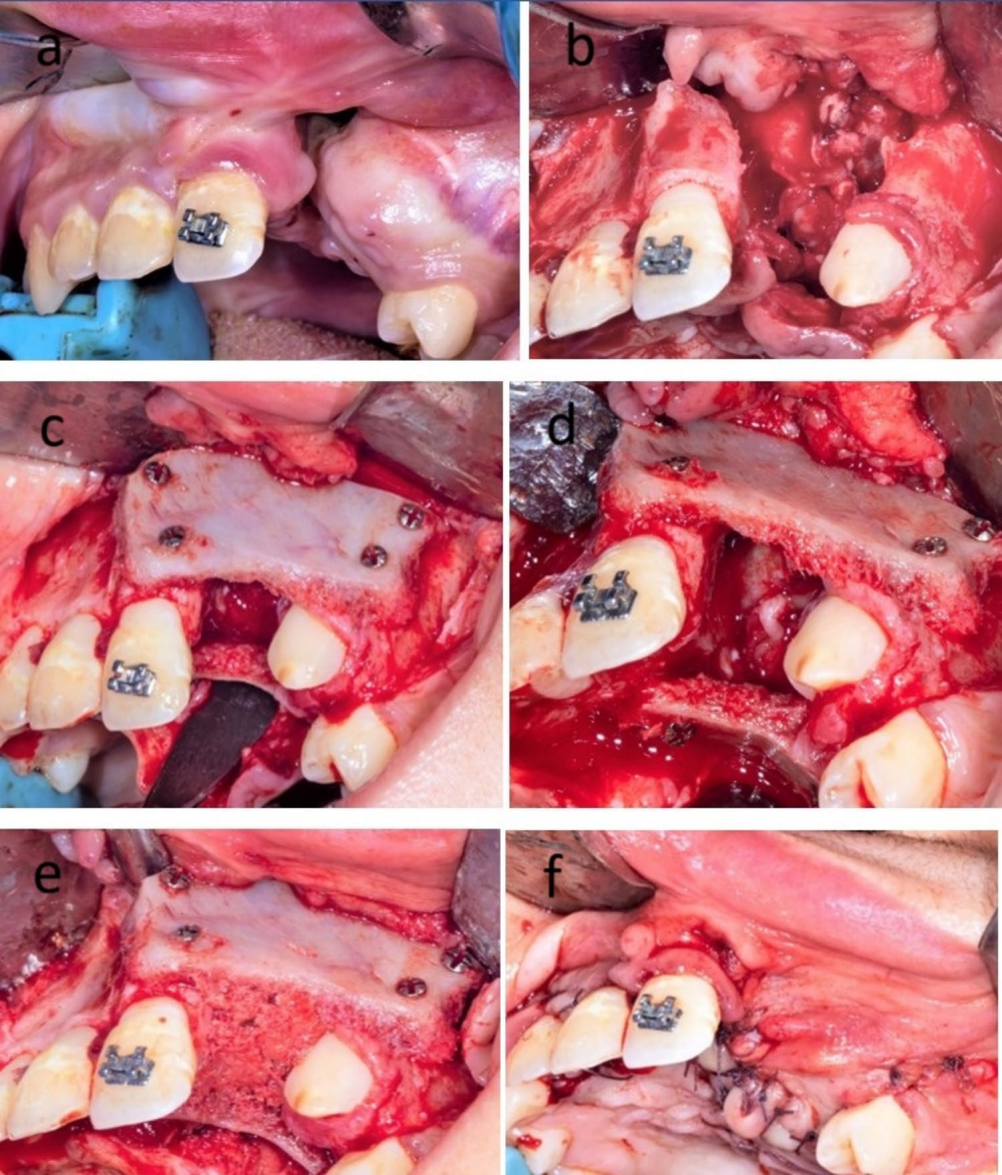
Clinical photographs showing alveolar cleft grafting using double iliac corticocancellous bone blocks with cancellous bone particles in between (Study group)

**Fig. 4:**
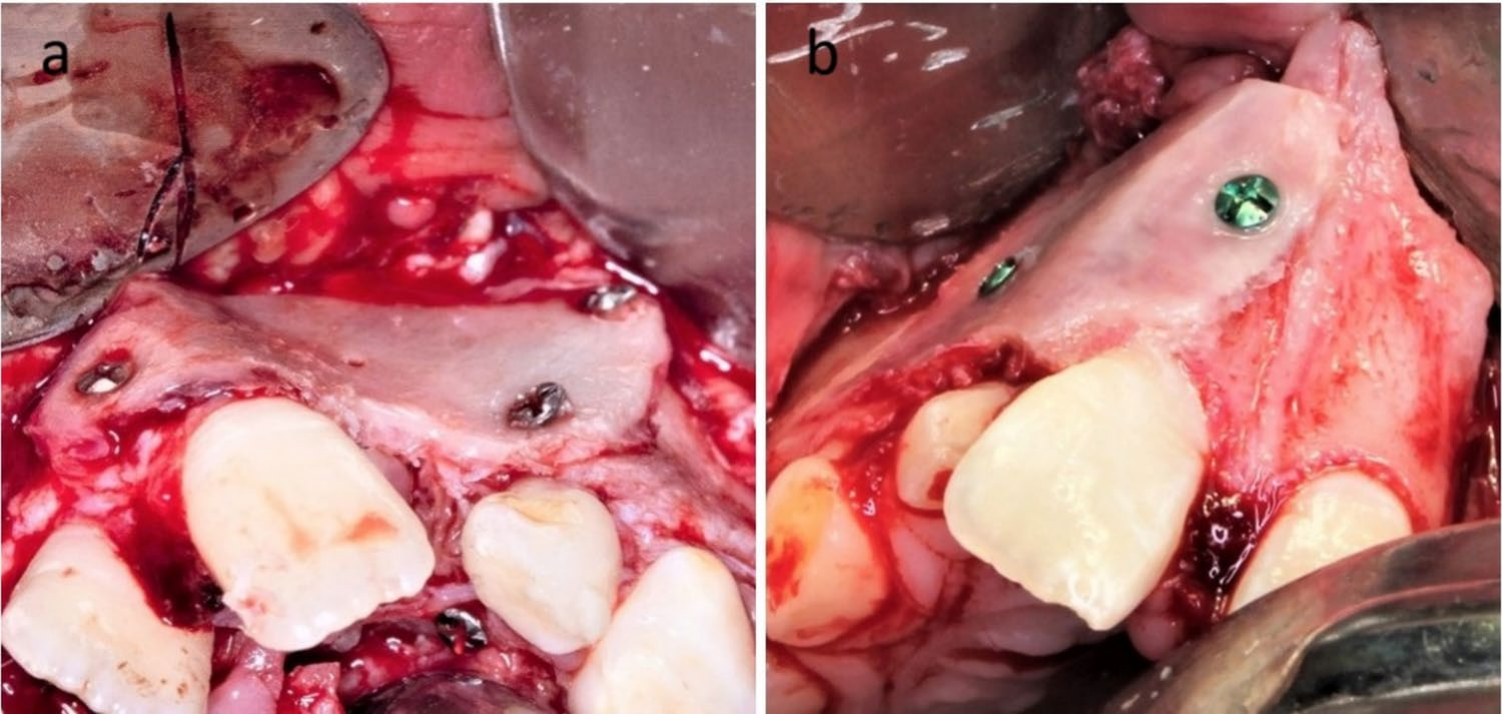
Two different iliac cortico-cancellous bone blocks bent, adapted, and fixed to the maxillary segments (Study group)

The harvested bone block was then divided into two corticocancellous bone blocks according to the size of the defect that was determined using a pattern (Fig. 2). The prepared bone blocks were large enough to overlap 3 to 5 mm of sound bone of both maxillary segments at both sides of the cleft margins. One of those bone blocks was fixed to the labial side of the cleft using 2 to 4 micro-screws to reconstruct the labial aspect of the cleft, while the other bone block was fixed usually with two screws palatal to the cleft and the gap in between filled with the harvested cancellous bone (Fig. 3). The micro-screws that were used were self-tapping and self-drilling screws that facilitated their fixation without the use of drills and hence helped in the avoidance of unintentional drilling through the roots of the teeth that were adjacent to the fixed bone blocks. The elasticity and malleability of the iliac cortical block facilitated its bending and adaptation in cases with different positions and the different external anatomy of the two maxillary segments (Fig. 4). Finally, the flaps of the recipient site were approximated and sutured using Vicryl 4.0 (Polyglactin 910; Ethicon) with interrupted fashion making sure that the closure was tension-free.

#### Postoperative measures

For the first 48 h, the dressing was applied over the donor site and after that, the skin wound was washed daily with betadine and warm saline. The patients and their guardians were asked to apply icepacks over the area of the recipient site for 15 min twice per hour for the first 24 h postoperatively and to keep a soft diet for two weeks postoperatively. From the day after surgery and for the next two weeks, instructions were made to every patient and their guardians that the patient’s mouth should be rinsed thoroughly with chlorhexidine and warm saline. Antibiotic therapy ((Amoxicillin/clavulanic acid 625 mg) (Augmentin, GlaxoSmithKlein, Egypt) bid, in some cases dose was adjusted according to the patient’s weight and age) was started postoperatively and continued for 7 days. To control pain, a non-steroidal anti-inflammatory analgesic (Ibuprofen 200 ml syrup) (Brufen, Abott laboratories, Egypt) tid, was prescribed immediately post-operative and for three days for all patients unless otherwise contraindicated. And to control edema, besides cold packs for the first 24 h and hot fomentations starting from the day after, an antiedematous drug was prescribed Ambezim® (Ambezim® by “Sanofi”).

#### Postoperative follow up and clinical assessment

The patient was checked the day after the operation for any possible complications, and to confirm that the postoperative medications and hygiene instructions were strictly followed. The patient’s wound was observed one week after the surgery. Sutures (of skin and mucosa) were removed within two weeks after the surgery and the wound was checked for any signs of dehiscence and/or infection. In the first month, the patient was followed once each week, once every month for the next 4 months, and once every two months. Screws were removed after 9 months under local anesthesia for elder patients while for the younger ones who couldn’t tolerate such maneuver, the screws were removed under GA. In cases where one or more of the screws were in the path of the eruption of one of the cleft teeth, screws were removed after 4 months.

#### Postoperative radiographic assessment

CBCT scans by Planmeca ProMax 3D Classic (Planmeca, Finland) were ordered within the first week postoperatively and at 9 months postoperatively. Then, the data was converted to DICOM files “Digital Imaging and Communication in Medicine”, to be examined, and compared. DICOM files of the two post-operative scans were assessed using two specialized software programs; Atomica.ai® for primary outcome measurements and 3D Slicer (www.slicer.org) for secondary outcome measurements.

**Fig. 5:**
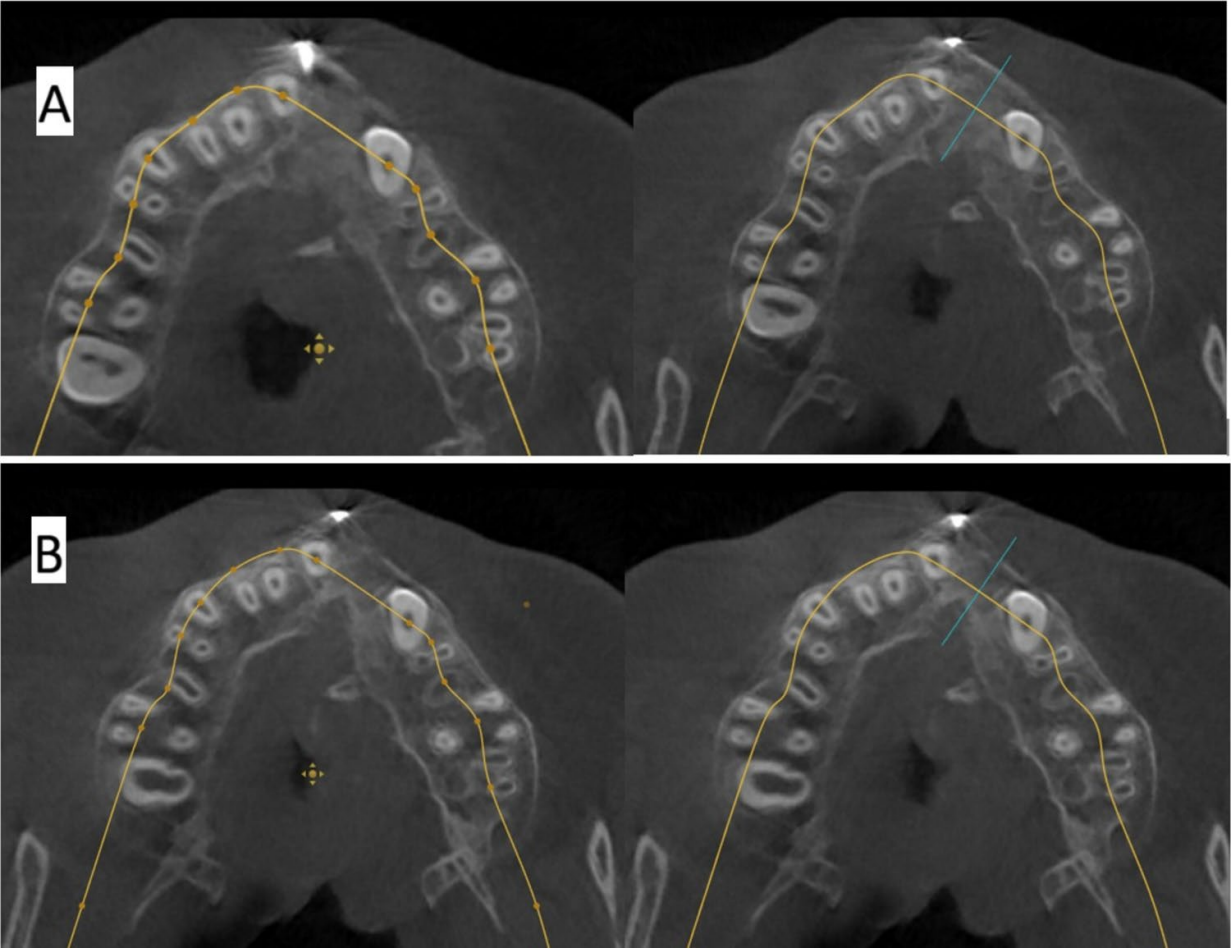
Photo-radiograph for standardization of **A)** immediately after surgery and **B)** 9 months post-operative width and height measurements for the study group

**Fig. 6:**
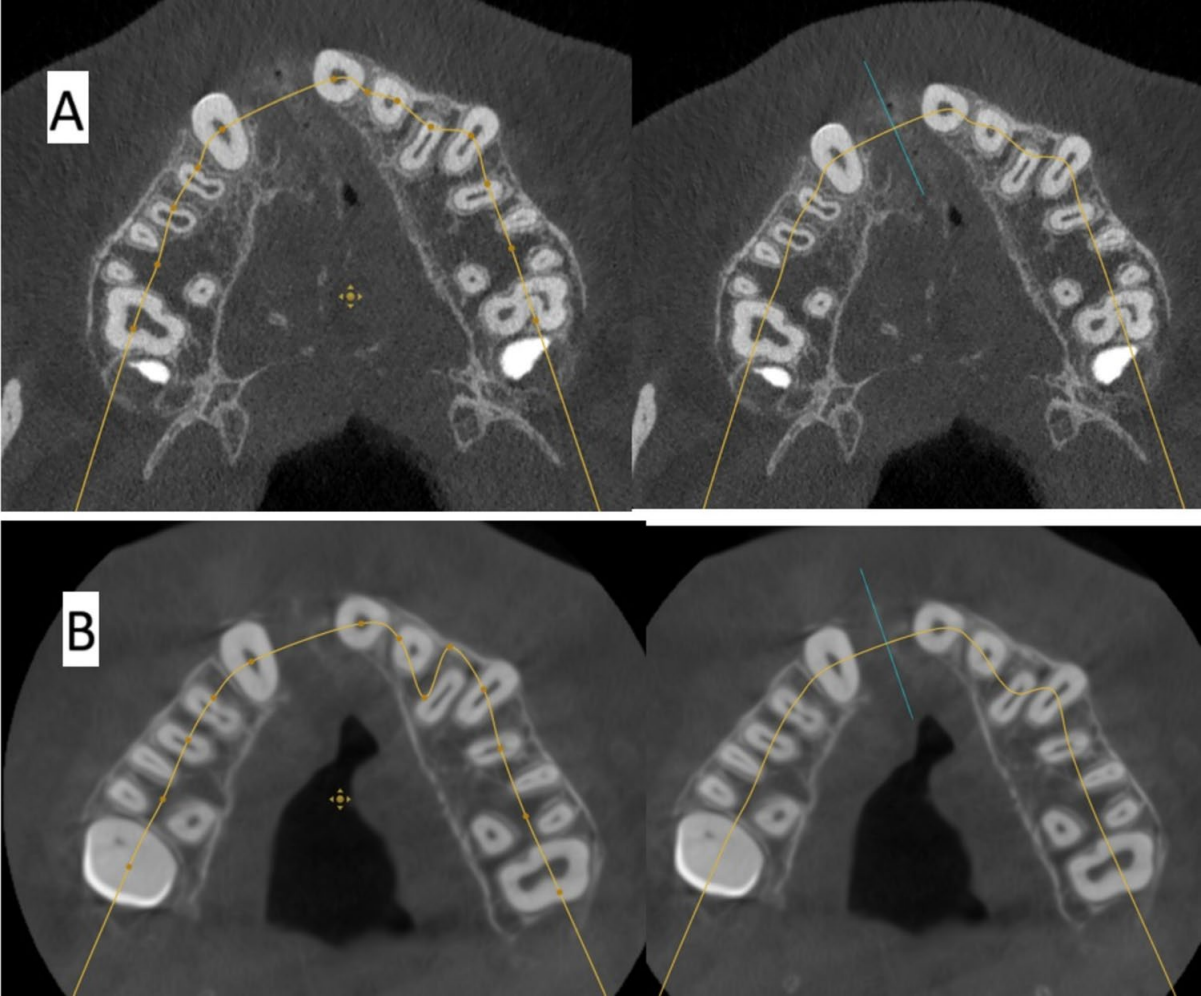
Photo-radiograph for standardization of **A)** immediately after surgery and **B)** 9 months post-operative width and height measurements for the control group

**Fig. 7:**
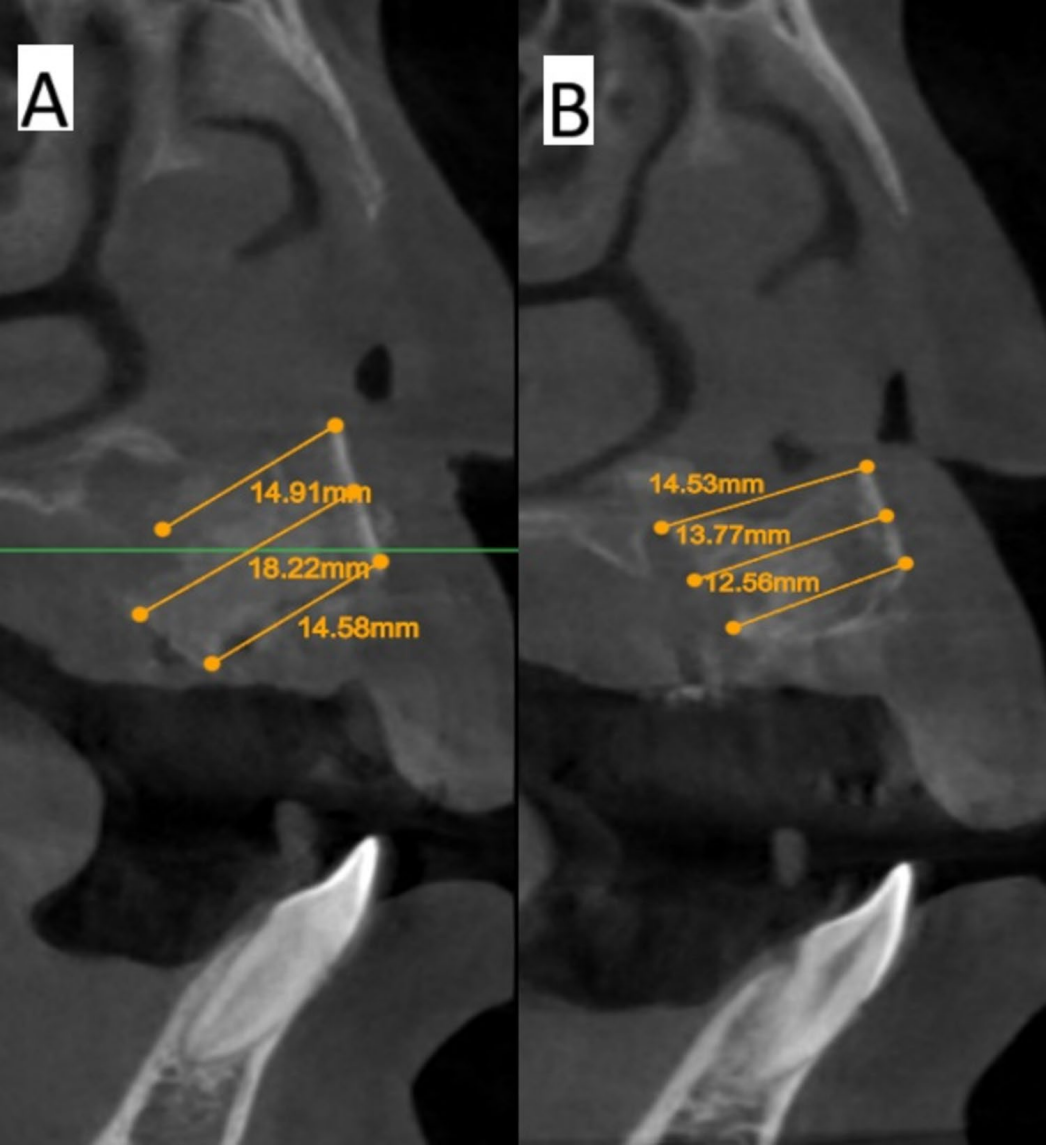
Photo-radiograph of **A) **immediately after surgery and **B)** 9 months post-operative labio-palatal width measurements for the study group

**Fig. 8:**
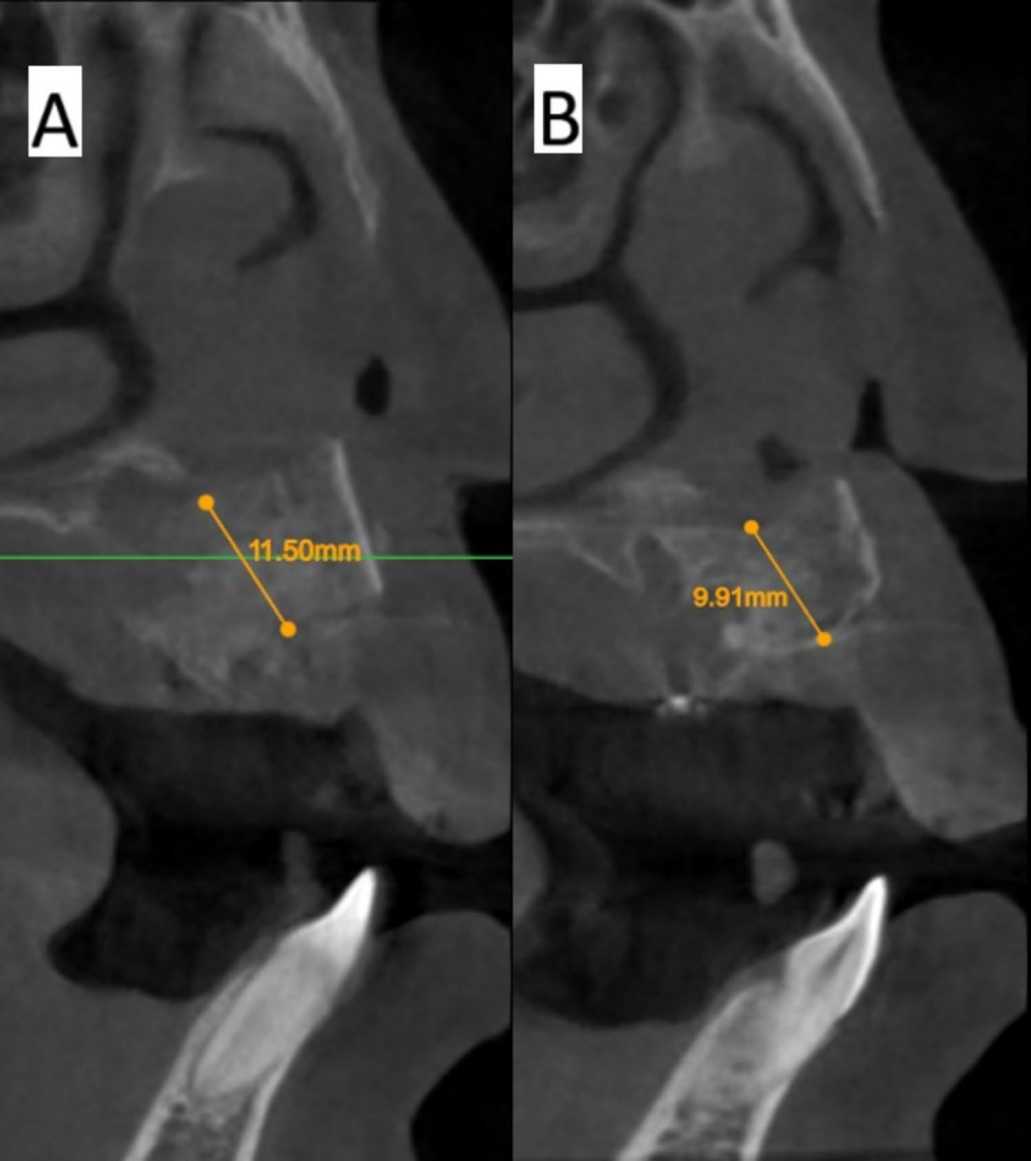
Photo-radiograph of**A) **immediately after surgery and **B)** 9 months post-operative height measurements for the study group

**Fig. 9:**
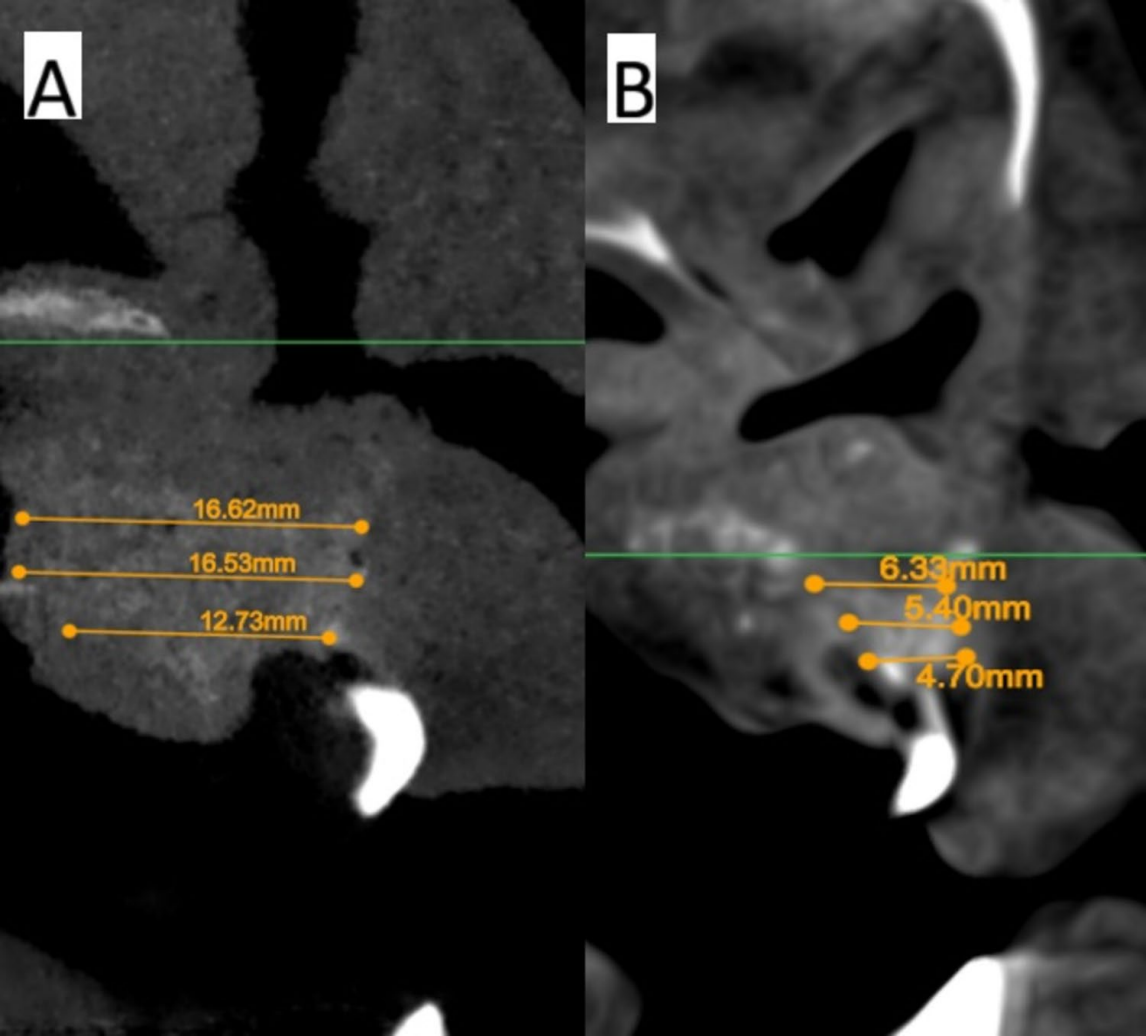
Photo-radiograph of **A) **immediately after surgery and **B)** 9 months post-operative labio-palatal width measurements for the control group

**Fig. 10:**
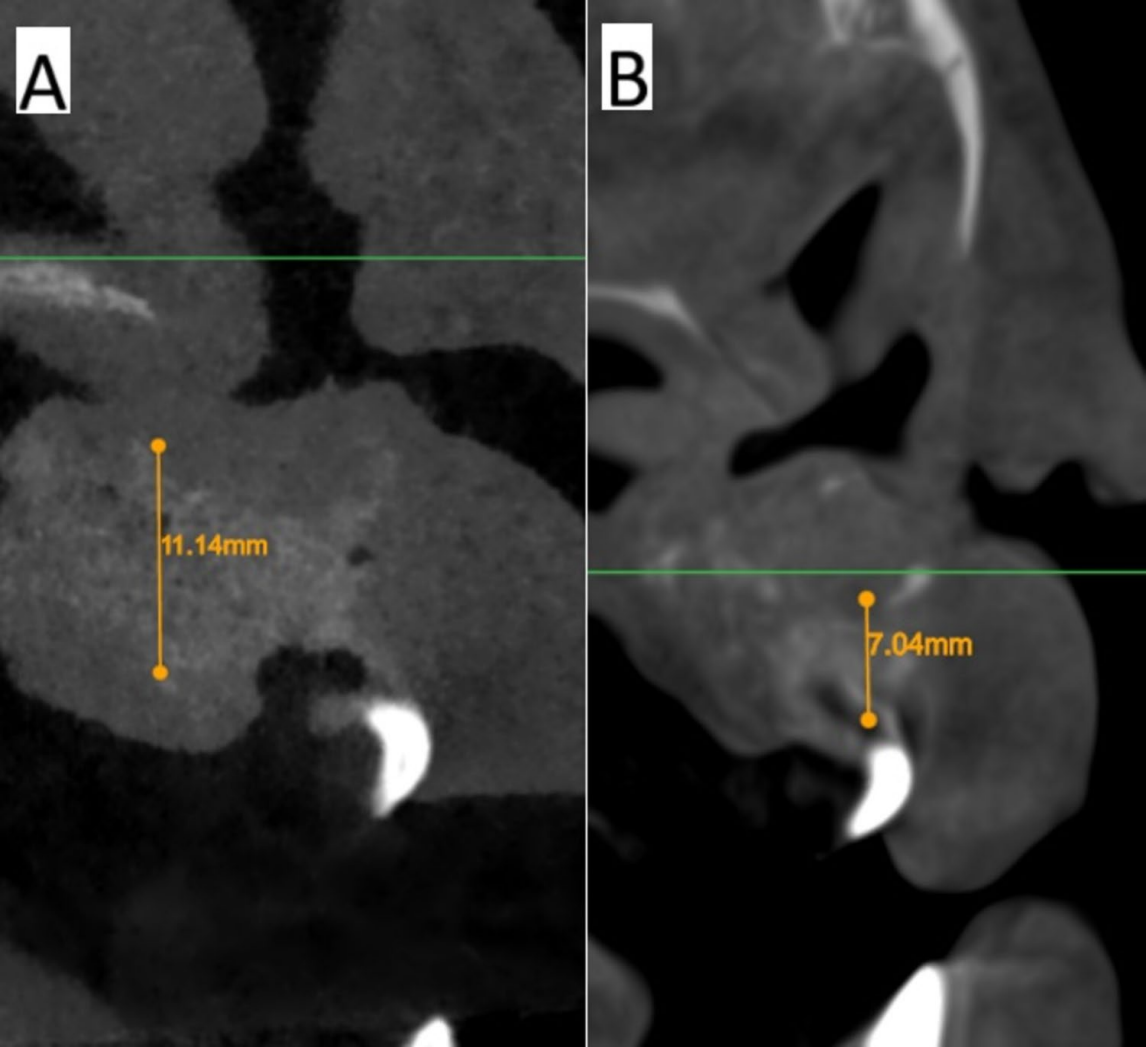
Photo-radiograph of **A) **immediately after surgery and **B)** 9 months post-operative height measurements for the control group

The software Atomica.ai ® is an advanced, free, and easy-to-use program for viewing and reformatting images developed by CBCT. It was used to assess the primary outcomes: graft height and labio-palatal width. After opening the DICOM file using Atomica.ai® software, the panoramic curve of the maxilla was generated automatically by choosing the maxillary curve and marking any number of teeth in the maxillary arch model. After that, the dots that form the curve were edited in the area of the teeth around the cleft making only two dots on both sides of the cleft/graft with each dot at the center of the root (pulp space) of each tooth at the level of cementoenamel junction making a straight line passing through cleft/graft area rather than a curved line which made the projections of the cross sections perpendicular to the graft area. Then, the projections were positioned at the center of the graft at a distance midway between the two roots of the cleft teeth (Figs. 5 and 6). Measurements of the labio-palatal graft width were made on 3 levels as follows: 1st level at the level of nasal mucosa/nasal floor (apical end of the graft), 3rd level at the level of CEJ of the central incisor (Coronal end/Crestal margin) of the graft, and 2nd level midway between both. Then, the average of those three measurements was calculated and recorded. The height of the graft was measured at the center of the graft by a line perpendicular to the three lines of measurements of labio-palatal width; this line extends from the nasal floor (apical end of the graft) to the crestal margin of the graft. Each group’s measurements were recorded, and comparisons between immediately after and after 9 months of measurements were made Figs. 7, 8, 9, and 10) [8].

**Fig. 11:**
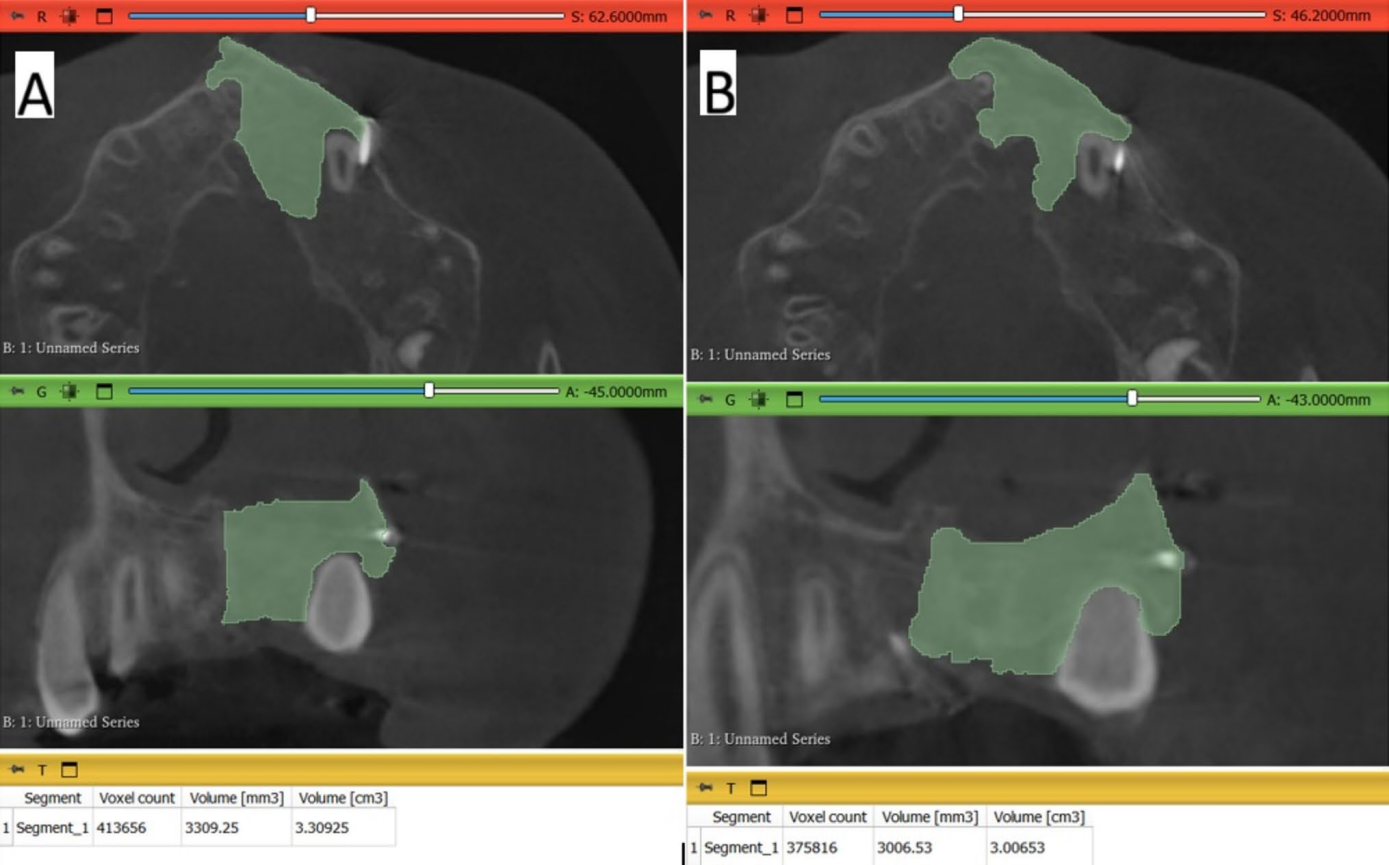
** A**) Photo-radiograph of immediate post-operative volume measurements for the study group.** B**) Photo-radiograph of 9 months post-operative volume measurements for the study group

**Fig. 12:**
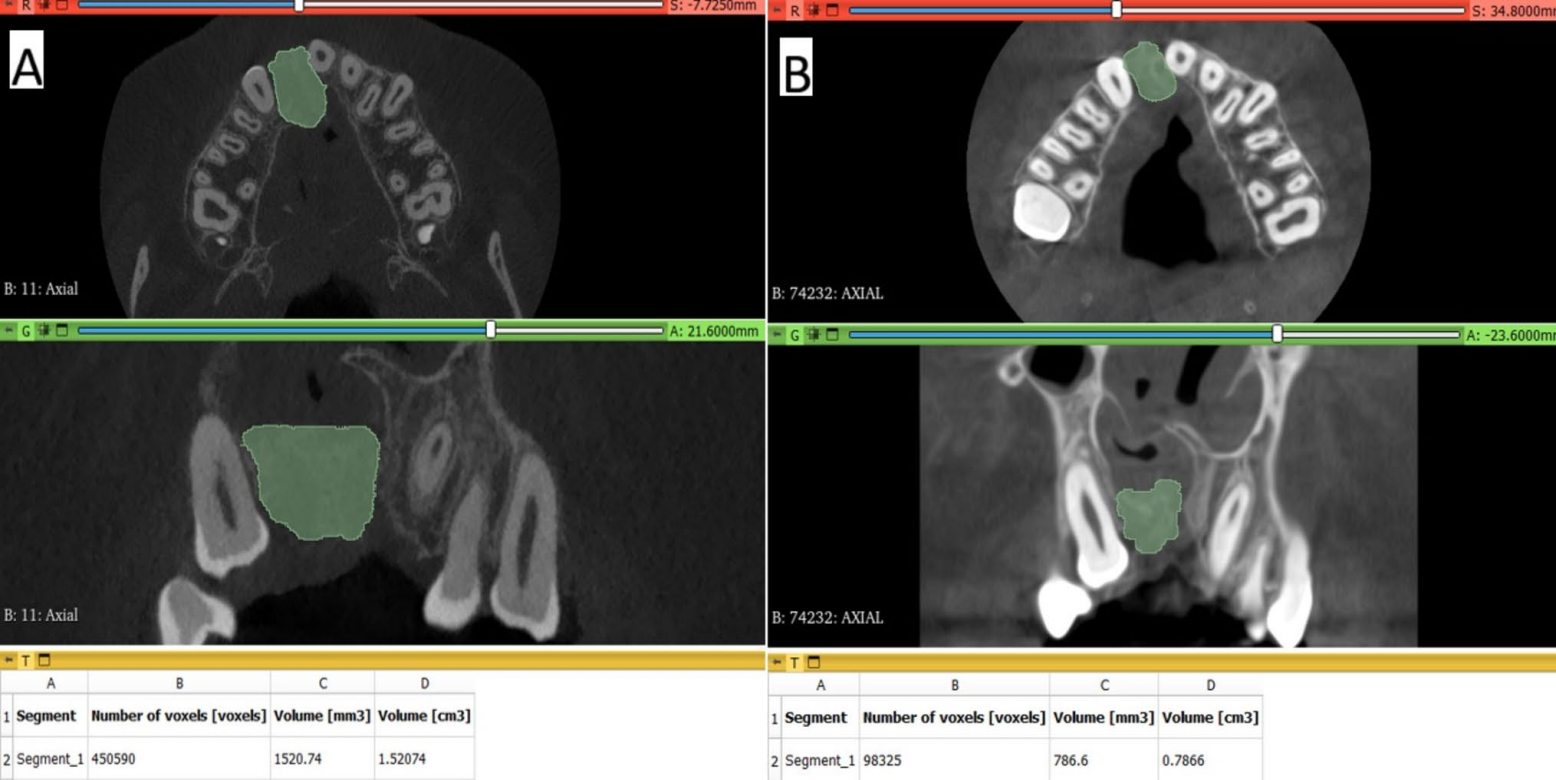
**A**) Photo-radiograph of immediate post-operative volume measurement for the control group. **B**) Photo-radiograph of 9 months post-operative volume measurement for the control group

The 3D Slicer (www.slicer.org), another free software for visualization, processing, segmentation, registration, and analysis of medical 3D images i.e.; CT and CBCT, was used in the current study to assess the secondary outcome; graft volume. ‘Segment Editor’ module was selected and the paintbrush tool was used to paint the graft slice by slice from the crestal margin of the graft to the level of the apical/nasal end of the graft and a single plane “Axial plane” in the full-screen window had been used as a start to easily identify the borders of the graft and refined by adding or erasing on each image of every other plane. Finally, the segmented/painted area’s volume was measured using the software's ‘Segment statistics’ module, automatically calculated in cm^3^ and mm^3^(Figs. 11 and 12 [8, 35]. Each group’s measurements were made 3 times by the same observer on the same day and recorded and then the mean measurements of each case of every group were used as a final record and comparisons between immediately after and after 9 months of volume measurements were made.

#### Statistical analysis

Analysis of categorical data provided as frequency and percentage values was made using Fisher’s exact test. The numerical data was presented as mean and standard deviation values. Their normality was explored by checking the data distribution and using the Shapiro–Wilk test. Normally distributed data was analyzed using an independent t-test for intergroup comparisons and repeated measures ANOVA followed by the Bonferroni post hoc test for intragroup comparisons. The non-parametric data was analyzed using the Mann–Whitney U test for intergroup comparisons and Friedman’s test followed by Nemenyi post hoc test for intragroup comparisons. Within all tests, the significance level was set at p < 0.05. R statistical analysis software version 4.3.1 for Windows (R Core Team (2023). R: A language and environment for statistical computing. R Foundation for Statistical Computing, Vienna, Austria. URL https://www.R-project.org) was used for statistical analysis.

An intra-class correlation coefficient (ICC) was used to measure the reliability of ratings (Test–retest reliability). The value of an ICC can range from 0 to 1, with 0 indicating no reliability and one is perfect reliability. According to Terry and Mae (2016) (Koo, T. K., & Li, M. Y. (2016). A Guideline of Selecting and Reporting Intraclass Correlation Coefficients for Reliability Research. Journal of chiropractic medicine 15(2), 155–163.) poor reliability (ICC less than 0.50), moderate reliability (ICC from 0.50 to 0.75), good reliability (ICC from 0.75 to 0.90), and excellent reliability (ICC greater than 0.90).

## Results

The study included 18 alveolar cleft patients who were equally and randomly allocated to each of the tested groups (9 cases each). The control group included 5 (55.6%) males and 4 (44.4%) females while in the intervention group, there were 4 (44.4%) males and 5 (55.6%) females. The mean age in the control group was (9.59 ± 2.00) years, and it was (9.33 ± 1.98) years in the intervention group, and the difference was not statistically significant (p = 0.789). Intergroup comparison and summary statistics for cleft volume (cm^3^) before surgery are presented in Table 1, which shows no significant difference between the two groups (p = 0.403).

**Table 1:** Intergroup comparison and summary statistics for cleft volume (mm3)

Time	Mean ± SD (cm^3^)		p-value
	Control	Intervention	
Before surgery	1.96 ± 0.63	2.28 ± 0.91	**0.403ns**

^*^; significant (p < 0.05) ns; non-significant (*p* > 0.05)

The healing of the donor site was uneventful for all cases of both groups except for a single case in the study group that presented with a small hematoma after 4 days which healed within two weeks with conservative measures only. Also, no changes in the walking gait in both groups was observed.

**Fig. 13:**
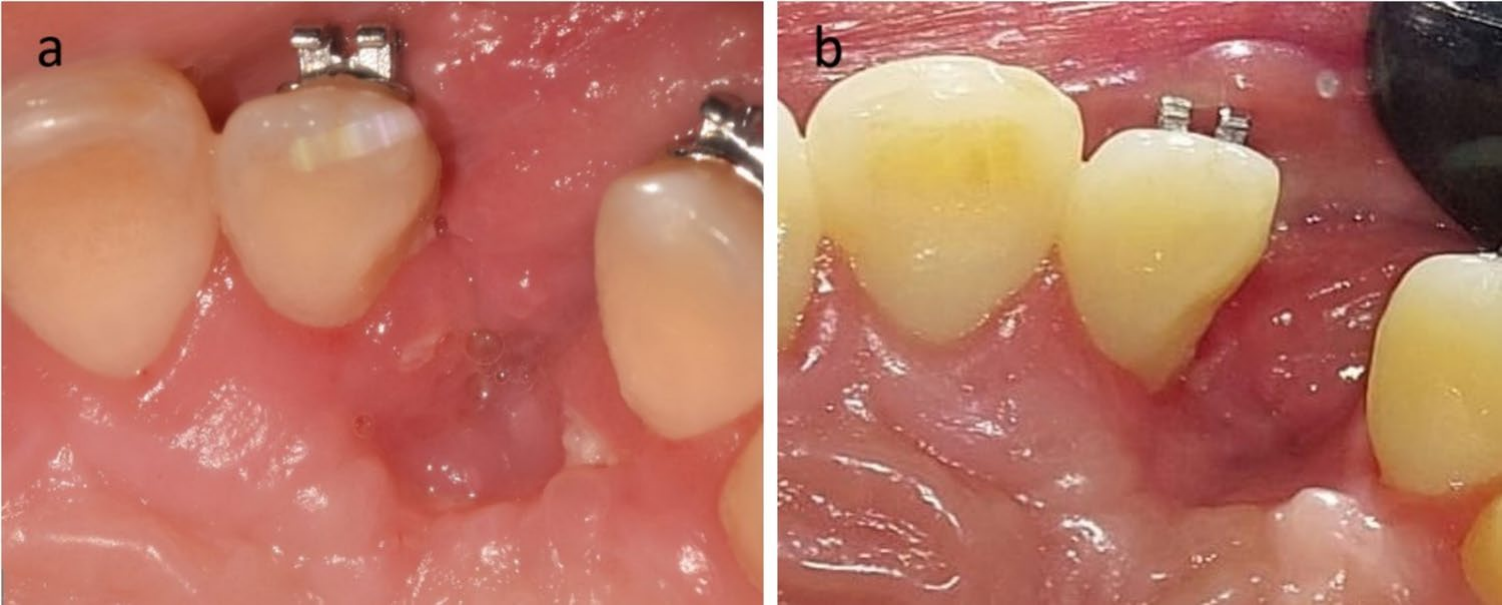
Case from study group showing **A**) Dehiscence of recipient site** B**) After healing

For the recipient site, in the control group, the healing was uneventful in all cases of the group, While the study group showed one case with a small dehiscence exposing a small area of the edges of the bone blocks which was treated by reducing the exposed bony margin using rose head carbide bur with copious irrigation and followed by strict oral hygiene measures. Gingival creeping occurred within two weeks, and complete healing was observed (Fig. 13).

The mean values for time of the surgery (minutes) in control group and intervention group were 157.33 ± 12.51 and 213.44 ± 10.34 min, respectively. The statistical analysis revealed there was significant difference (P < 0.05) in mean values of time of surgery (P = 0.0001) between control group and intervention group.

Immediately after surgery, there was no significant difference between both groups in terms of graft width which reached (12.53 ± 3.35 mm) in the control group and (13.17 ± 3.15 mm) in the intervention group (p = 0.685), while after 9 months graft width was significantly higher in the intervention group (11.42 ± 2.61 mm), while in the control group, the width was (7.07 ± 1.62 mm) (p < 0.001). Although the graft width showed a statistically significant decrease after 9 months (p < 0.05) for both groups, the difference in graft width between immediate postoperative measurements and after 9 months was significantly higher in the control group (5.47 ± 2.77 mm) (p = 0.002) while in intervention group the difference only reached (1.74 ± 1.23 mm). Inter, intragroup comparisons and summary statistics for graft width (mm) are presented in Table 2.

**Table 2:** Inter, intragroup comparisons and summary statistics for graft width (mm)

Time	Mean ± SD (mm)		p-value
	Control	Intervention	
Immediately after surgery	12.53 ± 3.35	13.17 ± 3.15	**0.685ns**
After 9 months	7.07 ± 1.62	11.42 ± 2.61	< 0.001*
p-value	< 0.001*	0.003*	
Difference	5.47 ± 2.77	1.74 ± 1.23	0.002*

^*^; significant (p < 0.05) ns; non-significant (*p* > 0.05)

For graft height measurements, inter, intragroup comparisons, and summary statistics for graft height (mm) are presented in Table 3. The results showed there was no significant difference between both groups in terms of graft height immediately after surgery, which reached (13.24 ± 2.16 mm) in the control group and (12.66 ± 1.86 mm) in the intervention group (p = 0.685), while after 9 months graft height was significantly higher in the intervention group (10.90 ± 1.38 mm), while in control group the height was (8.44 ± 1.63 mm) (p < 0.001). The difference in graft height between immediate postoperative measurements and after 9 months was significantly higher in the control group (4.80 ± 2.72 mm) (p = 0.002), while in the intervention group, the difference only reached (1.76 ± 0.75 mm).

**Table 3:** Inter, intragroup comparisons and summary statistics for graft height (mm)

Time	Mean ± SD (mm)		p-value
	Control	Intervention	
Immediately after surgery	13.24 ± 2.16	12.66 ± 1.86	**0.544ns**
After 9 months	8.44 ± 1.63	10.90 ± 1.38	0.003*
p-value	< 0.001*	< 0.001*	
Difference	4.80 ± 2.72	1.76 ± 0.75	0.005*

^*^; significant (p < 0.05) ns; non-significant (*p* > 0.05)

In terms of graft volume, immediately after surgery, there was no significant difference between both groups in terms of graft volume that reached (2.32 ± 0.91 cm^3^) in the control group and (2.42 ± 0.97 cm^3^) in the intervention group (p = 0.685), while after 9 months despite there was a significant decrease of graft volume for both groups, graft volume was significantly higher in the intervention group (2.08 ± 0.94 cm^3^) than in control group (1.14 ± 0.47 cm^3^) (p < 0.001). The difference in graft volume between immediate postoperative measurements and after 9 months was significantly higher in the control group (1.18 ± 0.53 cm^3^) (p = 0.002) while in the intervention group, the difference only reached (0.35 ± 0.15 cm^3^). Inter, intragroup comparisons and summary statistics for graft volume (cm^3^) are presented in Table 4.

**Table 4:** Inter, intragroup comparisons and summary statistics for graft volume (cm^3^)

Time	Mean ± SD (cm3)		p-value
	Control	Intervention	
Immediately after surgery	2.32 ± 0.91	2.42 ± 0.97	**0.815ns**
After 9 months	1.14 ± 0.47	2.08 ± 0.94	0.016*
*p*-value	< 0.001*	< 0.001*	
Difference	1.18 ± 0.53	0.35 ± 0.15	< 0.001*

^*^; significant (p < 0.05) ns; non-significant (*p *> 0.05)

In the current study, the ICC value for all variables in the control group and intervention group was more than 0.75 by the same observer/assessor (intra-observer reliability). The ICC for all variables in control group and intervention group were 0.776 (95% CI 0.482 – 0.940) and 0.853 (95% CI 0.660 – 0.961), respectively by the same assessor (intra-observer reliability). This indicated that acceptable variable measurements and a good reliability (P = 0.0001) for patients in both groups.

## Discussion

The conventional alveolar cleft grafting technique in which harvested cancellous bone particles from the anterior iliac crest are packed within the cleft space was considered the “gold standard”. This consideration was based on studies that judged the claimed success of the technique depending on scales and scores that usually used the graft height as a sole criterion for the success of the graft depending on 2D radiographs such as; occlusal or panorama x-rays. [8, 9, 11, 36].

Enemark found in a long-term study that; despite 90% of the patients showed successful results in the 2D radiographs, less than 50% of them could have successful orthodontic treatment in the area of the cleft, which means that the success of the conventional technique was highly overestimated. This is in agreement with a study by Lee et al. that stated that the dental radiograph significantly overestimated the number of clefts that could be managed orthodontically [36, 37].

So, the need for a 3D assessment of the graft seemed to be more reliable and informative. Van der Meij et al. study was one of the first studies to use CT scans to follow up the changes in graft volume after alveolar cleft grafting by conventional technique, and found that there was a mean graft volume reduction by about 30% in unilateral cleft cases, while in bilateral cleft cases, the reduction of graft reached about 55%. In another study by Honma et al., the volume of the graft by conventional technique measured using a CT scan after 1 year of grafting was statistically smaller than that measured after 3 months of grafting. Tai et al. followed the degree of graft volume reduction of the conventional SABG after one year, and there was an average of 43.1% reduction in total volume when assisted using a CT scan. In a study by Feichtinger who followed the changes in graft volume of the conventional technique using CT, found that the reduction of total graft volume reached 51% after one year of grafting and 52% after two years which is almost the same result that was reached in the current study’s control group that showed a mean reduction in graft volume after 9 months equal to 51%, while in the study group, the mean reduction in graft volume only reached 14.4% which reflects the greater stability of the graft through the healing period in the study group [9, 10, 13, 38].

In a study by Iino et al., the conventional grafting technique resulted in; only 48% of 29 cases showing acceptable labio-palatal thickness of the graft along the whole graft length, while the other cases suffered from different degrees of resorption of the labio-palatal thickness at the coronal, middle, and/or apical part/s of the graft. This is in agreement with the current study in which after 9 months there was a reduction in graft width by about 41.5% for the control group, while in the intervention group, the reduction in labio-palatal width reached only 13.9%. In the same study by Iino et al., when the height was measured there was an overestimation of the results of the conventional 2D radiographs when compared with CT results of the same cases. In another study by Omara et al. that used the same technique as the one used in the study group (the double iliac corticocancellous bone blocks technique) for alveolar cleft grafting, the average loss in bone height after 9 months was 1 mm, which is comparable to the current study in which the average bone loss in graft height for the study group reached about 1.76 mm, while in the control group, it was much higher and reached 4.8 mm [11, 33].

Studies by Feichtinger et al. and Oh et al. claimed that the presence of an actively erupting tooth at the graft site has a positive impact on graft volume maintenance, but in the current study, most of the cases in the control group had an actively erupting canine, yet there was a great amount of graft volume resorption after 9 months that reached 50.86% in average, and the graft healing was highly unpredictable despite the presence of actively erupting cleft tooth [13, 39].

However, one of the cases in the control group showed acceptable results with remarkably lower resorption, in which the alveolar cleft was incomplete with a palatal bony wall. The presence of a palatal wall against which the cancellous graft was packed had a great impact on the success of this case as it offered a larger surface area of the native bone surrounding and in contact with the graft, allowing for better graft stability and healing. This is in agreement with the basic principles of successful bone graft healing which include; stability of the graft and blood clot, angiogenesis, and space maintenance. Also, the palatal wall worked as a protective load and stress bearing against any external load that might fall upon the graft like food impinging on the mucosa covering the graft area during chewing, continuous pressure from the occlusal side by the contraction of the overlying deficient gingiva during healing and/or muscle pull from the soft tissue from the labial side due to scarred constricted lip of the patients. [13, 38, 39].

Similarly, the technique of the study group succeeded in maintaining the graft form, space, volume, and stability over the healing period due to the presence of the fixed two cortical bone blocks and their strong mechanical properties. The fixation of the bone blocks to the native bone using micro-screws gave stability to cleft segments and the particulate graft in between the fixed blocks. The obtained stability gave resistance against impinging food over the graft area and stability against the tension and contractile forces of the covering scarce soft tissue, hence preventing the collapse of the space of the graft and maintaining the graft’s volume during the healing period with only 14.46% average loss in graft volume [40–42].

Moreover, the bone blocks acted as a natural barrier, preventing soft tissue cells from creeping within the bone graft. This barrier mechanism allowed for the slow migration of the osteoblasts. The cancellous bone particles between the two corticocancellous bone plates helped in the rapid angiogenesis of the graft, rapid incorporation, and healing and provided a suitable medium for the active eruption of the cleft tooth [33, 42]. Despite the differences in the nature of the defects, source of the graft, and the patients age group, but this technique follows the same principles and comparable to the technique of the bone shells that is also called Khoury technique for the treatment of other alveolar defects in elder patients utilizing double mandibular cortical bone blocks [43].

The iliac bone blocks in such young age showed high degree of elasticity and bendability which allowed its fixation to the different morphologies and positions of the segments of the maxilla.

Pre-surgical orthodontics was initiated to cases from both groups for leveling of severely misaligned central incisors to allow for better access for placement of the graft and closure of the soft tissue. This was in agreement with the systematic review presented by Li Ma et al. [44].

One of the drawbacks of the technique of the study group is the relatively extended surgery time compared to the conventional technique, but it is still understandable due to increased number of maneuvers required to perform the surgery like, the medial approach to the anterior iliac crest, the harvesting of the corticocancellous bone block and its shaping, fixation, and trimming, however the higher success rate of the technique compared to the conventional one as was found in the current study, spares the patients from another grafting surgery with its consequences and morbidity.

The main complication in the study group was minor graft exposure, which might have been caused by graft overfilling and the rigid nature of the margins of the bone blocks, which had caused a dehiscence of the soft tissue that was already deficient. The treatment was done by reduction of the exposed cortical margin by the mean of carbide round bur with copious saline irrigation, and the overlying mucosa was allowed to heal by secondary intention. Only one case (11.11%) of the study group suffered from this minor dehiscence, but the healing after two weeks was uneventful and the effect of the removal of the exposed part of the graft didn’t affect greatly the total graft volume after healing. Even though the percentage of cases suffering from dehiscence is higher than that of the study by Van Nhan et al. (9.4%), it was less than that of the study by Omara et al. (16.6%). Also, the percentage of dehiscence in the current study was slightly lower than the assumption of dehiscence percentage after corticocancellous cleft grafting in a systematic review by Ma et al. (16%). This difference is believed to be due to the different sample sizes of the study groups [30, 33, 45]. One of the limitations of the intervention technique of the study group is the need for a second surgery to remove the fixative screws. The removal was done under local anesthesia for elder patients while for the younger ones who couldn’t tolerate such maneuver, the screws were removed under GA, usually after 9 months after the last follow-up CBCT. However, according to the results of the current study, when weighting the benefits and drawbacks of the technique of the intervention group, it was found less invasive to remove fixation screws even under GA than a whole regrafting surgery in case of the higher resorption rate of the conventional technique.

The other limitation of the current study is the small number of the cases, and so it is recommended to perform other RCTs with larger sample size to evaluate the efficacy and limitations of the technique of the intervention group compared to the conventional one.

## Conclusion

Regarding the graft success factors, which are represented by the stability of graft width, height, and volume over the follow-up period, the double iliac corticocancellous bone blocks with cancellous bone in between technique is markedly effective in reconstructing the alveolar cleft compared with the conventional autogenous iliac cancellous particulates bone graft.

## Data Availability

No datasets were generated or analysed during the current study.
